# Tracking lexical access and code switching in multilingual participants with different degrees of simultaneous interpretation expertise

**DOI:** 10.1111/ejn.15786

**Published:** 2022-08-09

**Authors:** Michael Boos, Matthias Kobi, Stefan Elmer, Lutz Jäncke

**Affiliations:** ^1^ Division Neuropsychology, Department of Psychology University of Zurich Zurich Switzerland; ^2^ Computational Neuroscience of Speech and Hearing, Department of Computational Linguistics University of Zurich Zurich Switzerland; ^3^ University Research Priority Program (URPP) “Dynamics of Healthy Aging” University of Zurich Zurich Switzerland

**Keywords:** auditory lexical decision, language switching, N400, speech perception

## Abstract

With the worldwide increase in people speaking more than one language, a better understanding of the behavioural and neural mechanisms governing lexical selection, lexical access in multiple languages and code switching has attracted widespread interest from several disciplines. Previous studies documented higher costs when processing a non‐native (L2) than a native (L1) language or when switching from L2 to L1. However, studies on auditory language reception are still scarce and did not take into account the degree of switching experience. Accordingly, in the present study, we combined behavioural and electrophysiological measurements to assess lexical access in L1 and L2 as well as code switching in professional simultaneous interpreters, trainee interpreters, foreign language teachers and Anglistics students, while the participants performed a bilingual auditory lexical decision task. The purpose of this study was to expand the knowledge on code switching in auditory language processing and examine whether the degree of simultaneous interpretation experience might reduce switching costs. As a main result, we revealed that L2 compared to L1 trials, as well as switch compared to non‐switch trials, generally resulted in lower accuracies, longer reaction times and increased N400 amplitudes in all groups of participants. Otherwise, we did not reveal any influence of switching direction and interpretation expertise on N400 parameters. Taken together, these results suggest that a late age of L2 acquisition leads to switching costs, irrespective of proficiency level. Furthermore, we provided first evidence that simultaneous interpretation training does not diminish switching costs, at least when focusing on lexical access.

## Graphical abstract

Language switches in the auditory domain lead to reduced accuracies, increased reaction times and larger N400 mean amplitudes—even in highly proficient individuals. Experience in simultaneous interpretation did not reduce language switching costs, at least in a lexical access scenario.

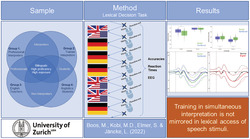

**Abbreviations**
BIA: Bilingual Interactive Activation model
E: English Trial
EEG: Electroencephalography
ERP: Event‐Related Potential
G: German Trial
IC: Inhibitory Control model
IntPro: Group of Professional Interpreters
IntStu: Group of Trainee Interpreters
L1: mother tongue (first language)
L2: second language
MulPro: Group of English Teachers
MulStu: Group of Anglistics Students
RTs: reaction times
SIs: simultaneous interpreters
WAIS: Wechsler Adult Intelligence Scale
ZHAW: Zurich University of Applied Sciences

## 1 INTRODUCTION

To speak more than one language is becoming more and more common worldwide. In fact, according to the last statistics of the European Commission, more than 60% of the population aged between 25 and 64 years can speak at least one additional language (L2) next to their mother tongue (L1). 1 Accordingly, the increasing population of individuals controlling two or more languages has attracted wide interest in neurocognitive research focusing on multilingual processing, language control and code‐switching (i.e., Abutalebi & Green, 2007; Fabbro, 2001; Mechelli et al., 2004; Perani et al., 1998; Rodriguez‐Fornells et al., 2009). The handling of multiple languages is a complex and multifaceted process that is far from being completely understood and involves both linguistic (i.e., Costa et al., 2006; Costa & Caramazza, 1999; Costa & Sebastián‐Gallés, 2014) and cognitive (i.e., Abutalebi & Green, 2007; Diamond, 2010; Lehtonen et al., 2018; Rodriguez‐Fornells et al., 2009; Sullivan et al., 2014) functions. This branch of research is further complicated by the fact that the cortical organization of multilingual processing has previously been shown to be influenced by several biographical variables, including age of L2 acquisition, level of L2 proficiency, L2 usage and exposure in everyday life as well as language switching experience (i.e., Consonni et al., 2013; Perani & Abutalebi, 2005; Perani et al., 2003).

One topic of particular interest in the field of bilingualism is the question of how lexical access in the two languages takes place. Two main frameworks have been proposed in this context, namely, a language selective and a language non‐selective perspective (Wang et al., 2020). While advocates of the language selective view assume that any linguistic stimulus activates only the respective language network, proponents of the language non‐selective lexical activation model believe that a linguistic stimulus can lead to the simultaneous activation of both languages. Drawing on this background, the language selective view implies the existence of independent lexica for the different languages. In contrast, the language non‐selective view relies on the assumption of a holistic bilingual lexicon (Dijkstra & van Heuven, 2002). Nevertheless, according to Wang et al. (2020), it seems that in both the visual (Dijkstra et al., 1999, 2000; van Assche et al., 2009; Van Hell & Dijkstra, 2002; Zhou et al., 2010) and the auditory domains (Grosjean, 1988; Pallier et al., 2001), there is more evidence in favour of the language non‐selective compared to the selective model. Furthermore, it is noteworthy to mention that besides the diverging conceptual frameworks discussed above, lexical access is also influenced by different biographical variables, including the age of language acquisition (Canseco‐Gonzalez et al., 2010; Hernandez & Li, 2007), the level of proficiency (Kastenbaum et al., 2019) and the degree of exposure (Bice & Kroll, 2016; Linck et al., 2009). Interestingly, there is currently also evidence that the phonotactic similarity between the spoken languages might influence lexical access (Kastenbaum et al., 2019). In auditory bilingual situations, lexical access must occur over time. Therefore, from a language non‐specific point of view, the activation of the bilingual lexicon is affected by phonetic features of the language input that guide lexical access (Wang et al., 2020).

A fundamental aspect that distinguishes bilinguals from monolinguals is that the former are not only able to effortlessly process different languages based on their phonological, lexical, semantic and syntactic abilities but also to dynamically switch between them (i.e., code‐switching). Notwithstanding these linguistic advantages, previous studies have shown that code switching is often associated with a performance cost that is manifested in longer reaction times and lower accuracies during switch compared to non‐switch trials in different kinds of tasks, including semantic categorization, numeral and picture naming, reading and lexical decision (i.e., Hut et al., 2017; Meuter & Allport, 1999; Mosca & de Bot, 2017; Price et al., 1999; Thomas & Allport, 2000; von Studnitz & Green, 1997). However, it is noteworthy to mention that switching costs also seem to differ between the different tasks mentioned above and between the domains of language production or comprehension (Mosca & de Bot, 2017). Furthermore, several previous studies have documented code‐switching asymmetries that were more or less pronounced depending on whether the tasks involved a change from L1 to a mastered L2 or vice versa. For example, in the late 90s, Meuter and Allport (1999) as well as other authors (e.g., Costa & Caramazza, 1999) showed that naming numbers or pictures in L1 after an L2 trial was associated with longer reaction times compared to naming items in L2 after an L1 trial. One possible interpretation of such switching asymmetries is that during L2 processing, the dominant L1 has to be more strongly inhibited to avoid interferences (Costa & Santesteban, 2004; Green, 1986, 1998; Meuter & Allport, 1999). This interpretation corresponds to the ‘Inhibitory Control’ (IC) model of Green (Green, 1986, 1998). In stark contrast, the ‘Bilingual Interactive Activation’ (BIA) model (Dijkstra & van Heuven, 1998; Grainger & Dijkstra, 1992; van Heuven et al., 1998) predicts higher costs when switching into L2 compared to L1. The reasoning behind this idea is that a word triggers more phonologically similar words in the lexicon of L1, leading to a higher inhibition of L2. Overcoming this inhibition is costly, and therefore, a switch from L1 into L2 is costlier than a switch from L2 into L1. Furthermore, switching costs have been shown to be dependent upon the degree of L2 proficiency, with higher costs in individuals with a low level of L2 mastery. In contrast, in fully balanced bilinguals, such asymmetric switching costs seem to decrease or even vanish (Costa & Santesteban, 2004). Finally, there is also evidence that code‐switching costs are modulated as a function of the tasks used. For example, switching costs usually are smaller when participants are asked to decide whether a letter string is a word or pseudoword, irrespective of language, compared to a situation where they have to judge if a stimulus is in a specific language (Jackson et al., 2004). Furthermore, processing costs tend to diminish if the switches are cued (Rogers & Monsell, 1995).

In a lexical decision task, participants are exposed to words and pseudowords that follow the phonotactic rules of a specific language and have to classify as quickly and accurately as possible whether each item is a real word or a non‐word. In such a context, a bilingual lexical decision task constitutes an interesting experimental approach to determine the influence of language expertise, L2 proficiency and age of language acquisition on lexical access in both L1 and L2 as well as on forward (L1‐to‐L2) and backward (L2‐to‐L1) code switching (Mimura et al., 1997). In this context, the degree and ease of lexical access are commonly assessed using accuracy metrics, reaction times or Event‐Related Potentials (ERPs). Results of several studies converge on the notion that words are recognized with higher accuracy and shorter reaction times than pseudowords, irrespective of modality (e.g., Barber et al., 2013; Friedrich et al., 2006; Krause et al., 2006; Mosca & de Bot, 2017). Furthermore, most of the electroencephalographic (EEG) studies using lexical decision tasks reported a more negative deflection of the N400 component for pseudowords in comparison to words (i.e., Barber et al., 2013; Bien et al., 2014; Friedrich et al., 2006; López Zunini et al., 2020) and to low‐frequency compared to high‐frequency words (i.e., Brysbaert et al., 2018; Monsell et al., 1989). The N400 component is commonly also more pronounced in switch compared to non‐switch trials (Hut et al., 2017; van der Meij et al., 2011), especially at parietal electrodes (van der Meij et al., 2011). Hence, from a neurolinguistics perspective, the N400 can be used as a salient marker of lexical access in that its amplitude increases with the processing costs of lexical‐semantic retrieval (Borovsky et al., 2010; Kutas & Federmeier, 2011). As proposed by the models introduced above, in a bilingual situation, the two languages compete for lexical‐semantic access, and the non‐target language has to be inhibited. Accordingly, when it comes to switching to a language that has been inhibited in the previous trial, the respective lexical‐semantic network has to be reactivated, resulting in increased N400 amplitudes (i.e., Hut et al., 2017; Pellikka et al., 2015). However, if a particular language node is already activated, no such reactivation is needed, and this should be manifested by smaller N400 amplitudes in within compared to between language trails. Finally, it is essential to mention that such switching costs are mainly observed in unbalanced bilinguals where L2 is explicitly controlled, and cognitive resources are needed to avoid interferences (i.e., DeLuca et al., 2019; Pliatsikas, 2020). In contrast, in highly proficient and balanced bilinguals, language control is more implicit and automated, resulting in lower switching costs. In this context, Hut et al. (2017) could show that a switch between two languages was not accompanied by additional costs in individuals with two mother tongues (i.e., simultaneous bilinguals).

The investigation of professional simultaneous interpreters (SIs) and trainee interpreters is particularly interesting because these individuals are situated at the higher end of the bilingual or multilingual continuum (DeLuca et al., 2019). Furthermore, this particular population of individuals is specifically trained to access the meaning of a source language while at the same time translating it into a target language under extreme time constraints (Elmer, 2012; Fabbro et al., 1991; Hervais‐Adelman et al., 2011). In this vein, SIs are highly trained in code switching and supposed to be characterized by a faster lexical‐semantic access (Elmer et al., 2010; Hervais‐Adelman et al., 2015). Even though the exact neural mechanisms underlying these exceptional language skills are somehow difficult to grasp, there is at least some evidence indicating training‐related plasticity effects in brain regions (Becker et al., 2016; Elmer, 2016; Elmer et al., 2014; Hervais‐Adelman et al., 2015; Hervais‐Adelman et al., 2017; Klein et al., 2018) and white matter pathways (Elmer et al., 2011, 2019; van de Putte et al., 2018) supporting several aspects of language processing as well as cognitive control mechanisms in general.

Currently, it is still a matter of debate whether such plasticity effects in SIs might facilitate lexical‐semantic processing and minimize switching costs. Nevertheless, such a beneficial influence of interpreting training on code‐switching mechanisms has been shown in non‐linguistic task‐switching paradigms where SIs demonstrated lower mixing costs than matched multilinguals (Babcock & Vallesi, 2017; Becker et al., 2016). In addition, in a language decision task where participants had to decide to which language a presented word belongs to, a language switch effect could only be found in bilinguals but not in SIs (Aparicio et al., 2017). Furthermore, taking into account forward and backward code‐switching at the sentence level, Proverbio et al. (2004) examined a group of professional SIs and provided evidence that switches into the trained language direction (usually from L2 into L1) resulted in shorter reaction times during reading and comprehension compared to the opposite direction. Finally, in a previous EEG study of our group (Elmer et al., 2010), we measured a sample of professional SIs and controls while the participants performed a semantic decision task consisting of judging whether auditorily presented noun pairs within and across languages (i.e., L1 = German, L2 = English) were either semantically congruent or incongruent. Results showed that SIs demonstrated larger N400 responses while processing incongruent trials both within L1 and L2 as well as while performing the task in the opposite direction as specifically trained (L1 to L2). Despite the results mentioned above, there is evidence indicating that adaptations in behaviour and brain responses in professional SIs are closely linked to their daily routines (i.e., Dottori et al., 2020; Hiltunen et al., 2016; Santilli et al., 2018). Taken together, these previous studies provided first evidence indicating that interpreting training might have an influence on lexical‐semantic processing within and across languages as well as on code‐switching mechanisms. However, a main drawback of these previous studies is that the tasks used often did not enable to disentangle lexical from semantic effects during both language processing and code switching. Furthermore, it is still unclear how the degree of language expertise, language proficiency, exposure and age of L2 acquisition influence lexical access and code‐switching during an auditory bilingual lexical decision task.

In the present study, we performed behavioural and electrophysiological measurements during an auditory lexical decision task to examine lexical access and code‐switching in different samples of multilingual participants who were highly proficient in the two tested languages (i.e., German and English), namely, professional SIs, SI students, foreign language teachers and students of English language and literature studies (Anglistics students). This study aimed to fill several gaps in the literature by addressing the following research questions. First, we wanted to replicate the asymmetric switching costs previously documented during picture naming, reading tasks and visual lexical decision tasks using an auditory lexical decision task. A second aim of the study was to test whether there are differences in lexical access within L1 and L2 as a function of language expertise and interpreting training. Furthermore, we examined whether individuals who are highly trained in code switching, namely, SIs, demonstrate comparable asymmetric switching costs as multilingual controls in a task requiring lexical access but not deeper semantic analyses.

## 2 MATERIALS AND METHODS

To provide a better overview of the sample characteristics and for the reader's convenience, we integrated the statistical analyses of the biographical, psychometric and linguistic data into Section 2 instead of reporting them separately in Section 3.

### 2.1 Participants

A total of 89 multilingual participants were recruited for this study (72 women and 17 men; Table 1). These participants were part of four different target groups, namely, trainee interpreters (IntStu; *n* = 30, 28 women [93%], mean age = 27.6, SD = 6.3), professional SIs (IntPro; *n* = 26, 21 women [81%], mean age = 46.9, SD = 12.3), Anglistics students (MulStu; *n* = 17, 15 women [88%], mean age = 23.6, SD = 5.3) and high school teachers of English (MulPro; *n* = 16, 8 women [50%], mean age = 39.8, SD = 13.1). IntStus and IntPros were recruited by collaborators of the Institute of Translation and Interpreting at the ZHAW (Zurich University of Applied Sciences). We used their network to contact SIs and interpreting students in Switzerland and Germany. MulStus were recruited at the universities of Zurich, Bern and Basel in Switzerland. In addition, we contacted all high schools in the German‐speaking part of Switzerland to recruit MulPros.

**TABLE 1:** Sample characteristics divided per group

Variable	N	IntStu, *N* = 30^a^	IntPro, *N* = 26^a^	MulStu, *N* = 17^a^	MulPro, *N* = 16^a^
Age (years)	89	28 (6)	47 (12)	24 (5)	40 (13)
Sex (proportion of females)	89	28 (93%)	21 (81%)	15 (88%)	8 (50%)
Handedness (dextral)	89	28 (93%)	24 (92%)	16 (94%)	14 (88%)
Age of L2 acquisition (years)	88	9.3 (4.3)	10.4 (3.8)	9.6 (3.8)	11.4 (2.7)
Language performance (x/40)	87	36 (2)	37 (2)	36 (2)	39 (1)
Cognitive capabilities (WAIS t‐score)	89	54.2 (5.5)	58.1 (4.4)	50.6 (4.8)	55.3 (4.2)
Interpretation training (h)	85	3,735 (8,828)	27,955 (19,363)	0 (0)	0 (0)
Interpretation performance (%)	87	0.62 (0.10)	0.76 (0.05)	0.59 (0.10)	0.64 (0.10)

*Note*: Missing data for some participants resulted in a smaller *N* than 89.

Abbreviations: IntPro, professional SIs; IntStu, SI trainees; MulPro, foreign language teachers; MulStu, Anglistics students.

^a^ Mean (SD); *n* (%).

Since matching the four groups in age was not possible due to the different degrees of expertise, we tried at least to match both student and professional groups that did not differ in this variable (IntStu vs. MulStu: *t*_(85)_ = 1.371, *p* = .521; IntPro vs. MulPro: *t*_(85)_ = 2.299, *p* = .106). The mother tongue (L1) of all participants was German, and English (L2) was learned at school at the age of about 10.05 years (SD = 3.82 years). Furthermore, the four groups did not differ in terms of age of L2 acquisition (*F*_[3,84]_ = 1.162, *p* = .329, η^2^_G_ = 0.04) Due to a technical issue, the data of one participant are missing. The primary interpretation direction for all professional SIs was from English (L2) to German (L1). As visible from Table 1, professional SIs had significantly more experience than all other three groups in simultaneous interpretation (*F*_[3,81]_ = 29.993, *p* < .001, η^2^_G_ = 0.526; due to a technical issue, the information from one participant is missing).

### 2.2 Questionnaires and psychometric measurements

Every participant completed an online questionnaire about their language background and performed a short English language test to verify L2 proficiency (sprachtest.de/einstufungstest‐englisch). The evaluation of the English test score revealed a significant main effect of ‘Group’ (*F*_[3,83]_ = 4.411, *p* = .006, η^2^_G_ = 0.138), and post‐hoc pairwise least‐square means analysis yielded a significant difference between MulPro and IntStu (*t*_[83]_ = −3.398, *p* = .006) as well as between MulPro and MulStu (*t*_[83]_ = 2.998, *p* = .018). In particular, the language teachers reached a higher score than both groups of students. Despite this significant effect, all groups reached a high score in the English test (IntStu: 36.4/40, SD = 2.31; IntPro: 37/40, SD = 2.49; MulStu: 36.4/40, SD = 2.06; MulPro: 38.7/40, SD = 1.35) underlining their good English proficiency. The total score of 40 points included a reading score (max. 5 points), a listening score (max. 7 points) and a vocabulary score (max. 28 points). For the analysis of the language performance, the data of two participants were missing due to a problem with the test provider's website.

A short version of the WAIS‐IV (Wechsler Adult Intelligence Scale) test battery was used to assess cognitive abilities (Waldmann, 2008). This procedure included four subtests, namely, number‐symbol associations, detecting commonalities, mosaic test and digit span forward and backward. This composition of subtests (i.e., standardized *T* values) has been shown to be sensitive to capturing general intellectual abilities (Waldmann, 2008). For our analysis, we gathered the mean of their normed values over all subtests to compute a resulting *T* score. Concerning these *T* scores in our sample, we revealed a significant effect of ‘Group’ (*F*_[3,85]_ = 8.376, *p* < .001, η^2^_G_ = 0.228). Post hoc analyses showed that IntPros scored higher than IntStus (*t*_(85)_ = 2.988, *p* = .019) and MulStus (*t*_(85)_ = 4.934, *p* < .001). In addition, MulPros showed a higher WAIS *t* score than MulStus (*t*_(85)_ = 2.734, *p* = .038).

All participants also completed the Annett questionnaire (Annett, 1970) to ensure that all groups were comparable concerning their handedness (*F*_[3,85]_ = 0.369, *p* = .775, η^2^_G_ = 0.013). Furthermore, since the stimuli of the lexical decision task were presented auditorily, all subjects performed a pure‐tone audiometry (ST20, MAICO Diagnostics, Berlin, Germany) to ensure a hearing acuity in the normal range (<40 dB). None of the participants reported suffering from psychiatric conditions, taking medication or suffering from neurological impairments. Participants were paid for participation, and written informed consent was obtained according to the declaration of Helsinki. The study was approved by the local ethics committee of the University of Zurich.

### 2.3 Interpretation rating

To confirm that the professional SIs were better at interpreting than the other three groups, we analysed the data of an additional interpretation task. During this task, the participants heard two different English conference speeches while fixating a cross presented on a screen. In this context, the participants had to covertly translate the speeches from English (L2) to German (L1) until the cross switched to a circle at a random point in time, indicating that the participants had to continue interpreting overtly. The cross and the circle switched multiple times in an irregular manner. This was done to get an EEG signal without muscular artefacts during the covert interpretation. Three raters scored the audio files of eight overt interpretation segments per participant using a custom unit‐by‐unit scoring template created by the chair of interpreting studies of the ZHAW (Zurich University of Applied Sciences), which is specialized in interpreting studies at the linguistic level. These templates enabled us to sub‐divide every sentence into smaller chunks that were classified into one of these categories: core (10), cohesion (9), meta‐discourse (7), secondary (5), modulation (3) or redundant (1). If the chunk was successfully interpreted, the rater gave points according to the numbers in brackets. With this procedure, each participant gained a total interpretation score that was used to compare interpreting performance between the groups. Since the three raters reached an intraclass correlation coefficient (ICC) of (C,1) = 0.89, inter‐rater reliability was almost excellent (Koo & Li, 2016). The analysis of the interpretation outputs according to the template described above revealed a significant main effect of ‘Group’ (*F*_[3,83]_ = 14.894, *p* < .001, η^2^_G_ = 0.35). In particular, as expected, professional interpreters scored better than interpreting students (*t*_[83]_ = 5.615, *p* < .001), Anglistics students (*t*_[83]_ = 5.788, *p* < .001) and foreign language teachers (*t*_[83]_ = 4.103, *p* = .001). Due to a technical problem with the microphone, two datasets were not properly recorded and therefore not included in the statistical analysis.

### 2.4 Lexical decision task and auditory stimuli

The auditory stimuli used for the bilingual lexical decision task consisted of 160 disyllabic German nouns (G, L1), 160 disyllabic English nouns (E, L2), as well as 80 disyllabic German and 80 disyllabic English pseudowords with a language‐specific phonotactic structure, resulting in a total of 480 stimuli (see Table S1 for a complete list). For every language, we selected 10 words belonging to one of 16 different categories like ‘vehicles’, ‘professions’, ‘vegetables’ or ‘birds’. The categories were chosen based on previous studies that evaluated objects' prototypicality of different semantic categories (Barbarotto et al., 2002; Maess et al., 2002). No translated versions of nouns or homophonic words like giraffe (E) /Giraffe (G) were used. According to the Corpora Collection of the University of Leipzig, word frequency ratios of the nouns were comparable for both languages (English: uk_web_2012, German: deu_newscrawl_2011). 2 In English, the average word frequency was 10.24, while in German, the respective average was 11.48. The word frequencies were compared between the two languages, and no difference could be found (*t*_[275]_ = −0.491, *p* = .624). Pseudowords were constructed for each language using the same syllables included in the real words. In particular, the syllables were paired in a random fashion, and afterwards, the resulting pseudowords were checked for phonetic plausibility to be a real word in the respective language. All stimuli were recorded in the phonetics laboratory of the University of Zurich by a female speaker. After recording, the stimuli were normalized and segmented into single units using the Adobe Audacity software (version 2.3.0). Stimulus presentation and the recording of behavioural responses during the lexical decision task were managed by the software ‘Presentation’ (version 20.1, Neurobehavioral Systems, Berkeley, USA, neurobs.com). Participants listened to the binaurally presented stimuli with on‐ear headphones (Sennheiser, HD 25–1 II, Ireland) at a sound pressure level of about 70 dB.

### 2.5 Experimental procedure

The psychometric measurements were conducted at the beginning of the experimental session. After having installed the EEG and completed the interpretation task, the lexical decision task was administered to the participants as the final part of the session. Participants were seated on a comfortable chair in a dimly lit room and faced a computer screen on which instructions were presented in black letters on a light grey background. A fixation cross was shown on the screen to minimize eye movements during the experiment. The computer mouse was used to collect the participants' responses, who were instructed to decide as fast and as accurately as possible if the heard stimulus was an existing word in either L1 or L2 (right click) or a pseudoword (left click). Before completing the task, a short test block of nine trials was presented to let the participants get a feeling for the procedure. Based on the previous stimulus, for each trial, we checked if it contained a language switch (i.e., EG and GE) or not (i.e., GG and EE). The stimulus sequence was then created pseudo‐randomly to ensure that every participant was exposed to 54 trials in each of the four conditions (EG, GE, GG and EE). For all statistical analyses, we only evaluated the second stimulus of a pair. The duration of each trial depended on the reaction time (RT) of the participants. In particular, after response selection, a jittered inter‐trial interval of 400–700 ms was inserted before the next stimulus was presented. Depending on the individual RTs, the lexical decision task had a duration of about 15 min. The participants did not receive feedback about their performance.

### 2.6 EEG recording

During the lexical decision task, continuous EEG was recorded with a sampling rate of 5,000 Hz. A total of 32 Ag/AgCl electrodes were applied according to the international 10‐10‐system (Fp1, Fp2, F7, F3, Fz, F4, F8, FT7, FC3, FCz, FC4, FT8, T7, C3, Cz, C4, T8, TP9, TP7, CP3, CPz, CP4, TP8, TP10, P7, P3, Pz, P4, P8, O1, Oz and O2), and a reference electrode was placed on the tip of the nose. Impedance levels were kept below 10 kΩ using abralyt gel. We used the EasyCap system manufactured by BrainProducts (BrainProducts, Munich, Germany).

### 2.7 Behavioural and EEG data preprocessing

**FIGURE 1:**
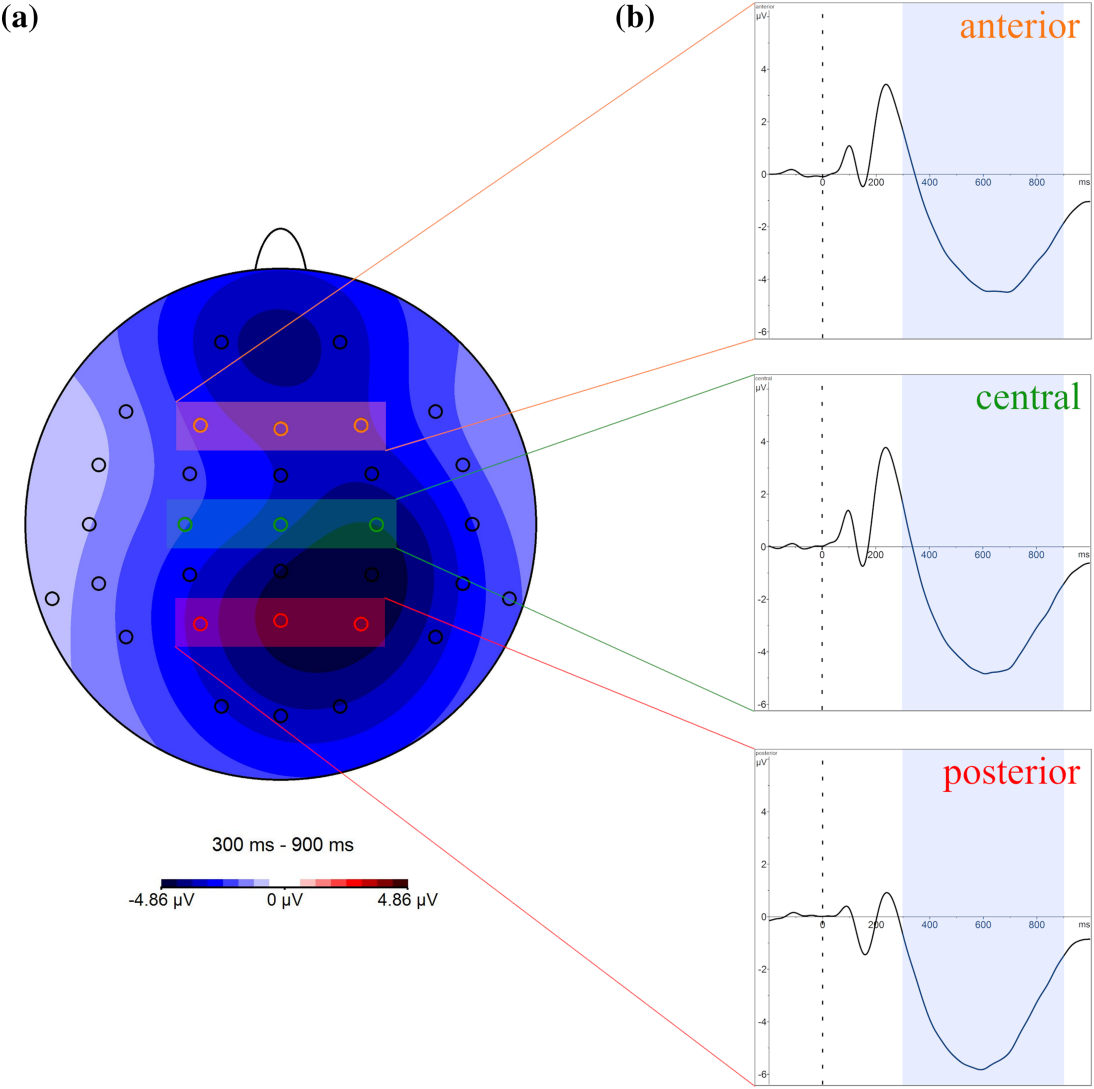
(a) The topographic voltage distribution of the Grand Average over all participants and all conditions is depicted. Marked are the electrode positions for the factor anterior–posterior (AP) at anterior (F3, Fz and F4; orange), central (C3, Cz and C4; green) and posterior (P3, Pz and P4; red) sites. The topographic voltage distribution covers the time window of 300–900 ms after stimulus onset. (b) Grand Averages over all participants and all conditions at the three AP sites. The time window of 300–900 ms after stimulus onset is marked in light blue.

Accuracy and RTs were analysed as behavioural data, and only correct trials were included in the RT analysis. Furthermore, to get rid of extreme values, only RTs longer or shorter than the lowest/highest quartile minus/plus 1.5 times the interquartile range were used for the analysis. In addition, extremely fast RTs below 250 ms were rejected (Ratcliff, 1993). In total, 4.2% of the trials were removed, most of them based on the upper boundary. EEG preprocessing was carried out in the Brain Vision Analyzer 2.0.2 (BrainProducts, Munich, Germany). First, a butterworth zero‐phase filter with a low cutoff at 0.1 Hz and a high cutoff at 20 Hz was applied to the data. Additionally, a Notch filter with the local power line frequency of 50 Hz was used. Artefact correction was done using a semi‐automatic independent component analysis (ICA) procedure (Jung et al., 2000) to remove saccade and blink artefacts. Subsequently, an automatic raw data inspection was performed to eliminate further muscle artefacts. Data were epoched into segments of 1,200 ms length, including a baseline from −200 ms to stimulus onset. Only correctly answered trials were included in the EEG analyses. In addition, similar to the aggregation of the RT data, only trials with responses above 250 ms and within the time range of the lowest/highest quartile minus/plus 1.5 times the interquartile range entered further analysis steps. The inclusion rate of trials per group was for IntStu: 0.940, SD = 0.0546; for IntPro: 0.956, SD = 0.0408; for MulStu: 0.933, SD = 0.0538; and for MulPro: 0.950, SD = 0.0539. This resulted in a mean of 50.8/54 trials included for IntStus, 51.6/54 for IntPros, 50.4/54 for MulStus and 51.3/54 for MulPros. Regarding the conditions, a mean of 50.8/54 trials entered analysis for the EE condition, 49.3/54 for the GE condition, 52.0/54 for the GG condition and 52.1/54 for the EG condition. There were no differences in the number of included trials per group (F_[3,85]_ = 1.738, *p* = .165, η^2^_G_ = 0.035). In contrast, and as expected because we included only correctly answered trials for further analysis of the EEG data, the number of included trials was higher for the German trials compared to the English ones (F_[1,85]_ = 78.712, *p* < .001, η^2^_G_ = 0.144) and higher for non‐switch compared to switch trials (*F*_[1,85]_ = 16.631, *p* < .001, η^2^_G_ = 0.020). However, more than 90% of the trials were used for the analysis in all conditions. Due to the interpretation task administered before the lexical decision task, the participants were already quite tired, resulting in increased muscular artefacts at frontopolar electrodes. Hence, to reduce the influence of these artefacts on ERP metrics, an additional low‐pass filter of 15 Hz was applied. Finally, the single segments of each of the four conditions were averaged, and mean amplitudes were extracted for every trial (i.e., GG, EE, GE and EG) in the time‐range corresponding to the N400 component, namely, between 300 and 900 ms after stimulus onset (Figure 1, marked in blue). Signed mean activity values in μV of the whole time window were extracted. The time window corresponding to the N400 component was chosen based on the grand average computed across all participants and conditions (Figure 1). Furthermore, we checked the topographies during the chosen time window to provide further evidence for common neural generators during the entire processing stage of the N400 component. In particular, based on the distribution of the N400 component along the anterior–posterior topographical axis (Figure 1) and in line with previous studies that evaluated the N400 component (Dittinger et al., 2019; Elmer et al., 2021; Erlbeck et al., 2014), mean N400 amplitudes were evaluated at an anterior (F3, Fz and F4), central (C3, Cz and C4) and posterior (P3, Pz and P4) pool of electrodes. Because the results of an additional frontopolar pool did not differ from those of the anterior pool, only the results of the anterior pool are described in more detail below. For further analysis, the peak latencies of the N400 components were extracted for each electrode pool separately.

### 2.8 Statistical analyses

All behavioural and electrophysiological data were evaluated using the R software package (version 3.6., www.r-project.org/), and all reported ANOVAs were calculated with the R package ‘ez’ (version 4.4, CRAN.R‐project.org/package=ez). An α = 0.05 was set as the default significance level used for all analyses—unless stated otherwise. If sphericity was not met, *p* values and degrees of freedom are reported using a Greenhouse–Geisser correction. For further post hoc tests, the R‐package ‘emmeans’ was used to generate pairwise comparisons of estimated marginal means between groups (version 1.5.4, CRAN.R‐project.org/package=emmeans). The package automatically corrects for multiple comparisons (Tukey method). All ANOVAs included the factors ‘Condition’ (two levels; Word and Pseudoword) or ‘Switch’ (two levels; Switch and NonSwitch), ‘Language’ (two levels; German and English) and ‘Group’ (four levels; IntStu, IntPro, MulStu and MulPro). For the analysis of the EEG data, an additional factor, ‘Anterior–Posterior’ (AP, three levels; ‘Anterior’, ‘Central’ and ‘Posterior’) was used. For all ANOVAs, the effect sizes are reported using generalized eta squared (η^2^_G_) because this measure has the advantage of good comparability across within‐ and between‐subjects designs (Bakeman, 2005). Based on the fact that the English test scores and the WAIS t‐scores differed between the groups, we added them as covariates in the statistical models. However, since both variables showed a negligible impact on the dependent variable, they were not included in the final analyses.

## 3 BEHAVIOURAL AND ELECTROPHYSIOLOGICAL RESULTS

### 3.1 Word‐Pseudoword effect

For both accuracy and RT measures the word‐pseudoword effect was evaluated by means of 2 × 4 ANOVAs with the factors ‘Condition’ and ‘Group’. Otherwise, for the EEG analysis, an additional ‘AP’ factor was added, resulting in a 2 × 4 × 3 ANOVA (2 Condition × 4 Group × 3 AP). The first ANOVA regarding accuracies yielded a main effect of ‘Condition’ (*F*_[1,85]_ = 71.279, *p* < .001, η^2^_G_ = 0.301) and a main effect of ‘Group’ (*F*_[3,85]_ = 4.423, *p* = .006, η^2^_G_ = 0.071). Only a main effect of ‘Condition’ was found for the ANOVA with RT data (*F*_[1,85]_ = 366.020, *p* < .001, η^2^_G_ = 0.515). Furthermore, the evaluation of the EEG data by means of a 2 × 4 × 3 ANOVA yielded a main effect of ‘Condition’ (*F*_[1,85]_ = 15.871, *p* < .001, η^2^_G_ = 0.019), a main effect of ‘AP’ (*F*_[2,170]_ = 21.961, *p* < .001, η2G = 0.036) and an interaction between ‘Condition’ and ‘AP’ (*F*_[2,170]_ = 141.519, *p* < .001, η^2^_G_ = 0.021).

The main effect of ‘Condition’ in the accuracy data revealed that words were detected more accurately than pseudowords, with a mean accuracy of 0.963 compared to 0.895. In addition, post hoc pairwise comparisons used to disentangle the significant main effect of ‘Group’ in the accuracy data revealed that the professional SIs were more accurate in their answers than both student groups (IntStu – IntPro: *t*_(85)_ = 2.811, *p* = .031; MulStu – IntPro: *t*_(85)_ = 2.55, *p* = .059). The evaluation of the accuracy data did not reveal a significant interaction between ‘Condition’ and ‘Group’. Furthermore, the evaluation of RTs revealed that words were detected faster than pseudowords with a mean RT of 951 ms (SD = 87.2 ms) compared to 1,219 ms (SD = 176 ms). Neither the main effect of ‘Group’ nor the interaction between ‘Condition’ and ‘Group’ reached significance.

The evaluation of the EEG data revealed significant main effects of ‘Condition’ and ‘AP’. In particular, words elicited smaller N400 amplitudes than pseudowords, and the post‐hoc comparison of the three electrode locations revealed significantly stronger negativities at posterior sites compared to anterior ones (*t*_[170]_ = 5.648, *p* < .001) and central ones (*t*_[170]_ = 4.767, *p* < .001). Finally, the omnibus ANOVA also revealed an interaction between ‘AP’ and ‘Condition’. To disentangle this interaction, we further compared the amplitude differences between the word and pseudoword conditions at all three pools of electrodes. This procedure yielded significant results at the central (*t*_[106]_ = −2.592, *p* = .011) and posterior (*t*_[106]_ = −8.324, *p* < .001) pools. The most pronounced difference between words and pseudowords was found at posterior electrodes with a difference of about −1.636 μV, whereas the difference at the central pool was of about −0.509 μV.

### 3.2 Code‐switching effects

**FIGURE 2:**
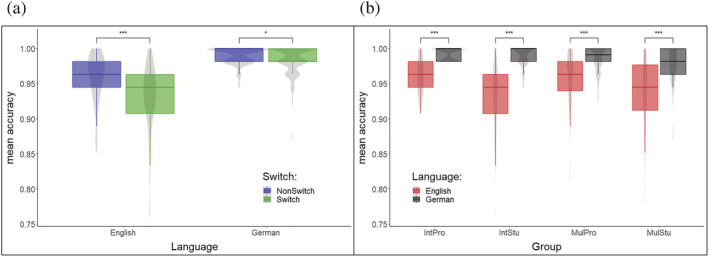
(a) Depiction of the ‘Switch’ X ‘Language’ interaction effect. As can be seen from the figure, switch (green) and non‐switch trials (blue) led to diverging accuracies in both languages. While in both German and English non‐switch trials were dealt with higher accuracy, the difference between switch and non‐switch accuracies was larger in English as compared to German. (b) Depiction of the ‘Group’ X ‘Language’ interaction effect. The mean accuracy values are shown separately for both the English (red) and German (black) languages and the four groups. While the accuracy difference between English and German was largest for IntStu, it was smallest for IntPro and MulPro. Abbreviations: IntPro, professional SIs; IntStu, SI trainees; MulPro, foreign language teachers; MulStu, anglistics students, ***p < .001, **p < 0.01, *p < 0.05

For the code‐switching effects, only the four conditions without pseudowords were analysed (EE, EG, GE and GG trials). The 2 × 2 × 4 ANOVA (2 ‘Language’ × 2 ‘Switch’ × 4 ‘Group’) computed on accuracy data yielded significant main effects of ‘Language’ (*F*_[1,85]_ = 164.274, *p* < .001, η^2^_G_ = 0.351), ‘Switch’ (*F*_[1,85]_ = 50.64, *p* < .001, η^2^_G_ = 0.093) and ‘Group’ (*F*_[3,85]_ = 5.588, *p* = .002, η^2^_G_ = 0.073) as well as an interaction between ‘Language’ and ‘Switch’ (*F*_[1,85]_ = 19.292, *p* < .001, η^2^_G_ = 0.033) and ‘Group’ and ‘Language’ (*F*_[3,85]_ = 4.906, *p* = .003, η^2^_G_ = 0.046). These effects are displayed in Figure 2.

The main effect of ‘Language’ was related to the fact that German words were recognized better than English ones (G = 0.987, E = 0.946), whereas the main effect of ‘Switch’ indicated that non‐switch trials were answered more accurately than switch trials (non‐switch: 0.976, switch: 0.958). Furthermore, the main effect of ‘Group’ originated from the fact that, compared to SI students (*t*_(85)_ = 3.349, *p* = .007) and Anglistics students (*t*_(85)_ = 3.369, *p* = .006), professional SIs performed significantly more accurately. To disentangle the interaction between ‘Language’ and ‘Switch’, we compared the accuracies for switch and non‐switch trials separately for both languages. According to this procedure, in both languages the accuracy values differed significantly between switch and non‐switch trials (English: *t*_(169)_ = 7.765, *p* < .001; German: *t*_(169)_ = 2.342, *p* = .020), and in the English language, this difference was larger as revealed by the comparison of the estimates of the contrasts stated above (*t*_(85)_ = 3.980, *p* < .001, Figure 2). Finally, the ‘Group’ X ‘Language’ interaction showed that primarily the IntStus were less accurate in the English trials compared to the German trials. No accuracy differences between groups reached significance when looking at the German trials. For the English trials, IntPro reached higher accuracies than IntStu (*t*_[165]_ = 4.562, *p* < .001) as well as MulStu (*t*_[165]_ = 3.016, *p* = .016), and MulPros reached higher accuracies than IntStu (*t*_[165]_ = 3.589, *p* = .002). As visible from Figure 2, the IntStu showed the most pronounced difference between the two languages, whereas this difference was smallest for the IntPro and the MulPro. This was also reflected by the results of the group‐wise comparison of the language differences. In fact, only the contrast between IntPro and IntStu (*t*_(85)_ = 3.108, *p* = .013) as well as the contrast between MulPro and IntStu (*t*_(85)_ = 3.282, *p* = .008) reached significance.

**FIGURE 3:**
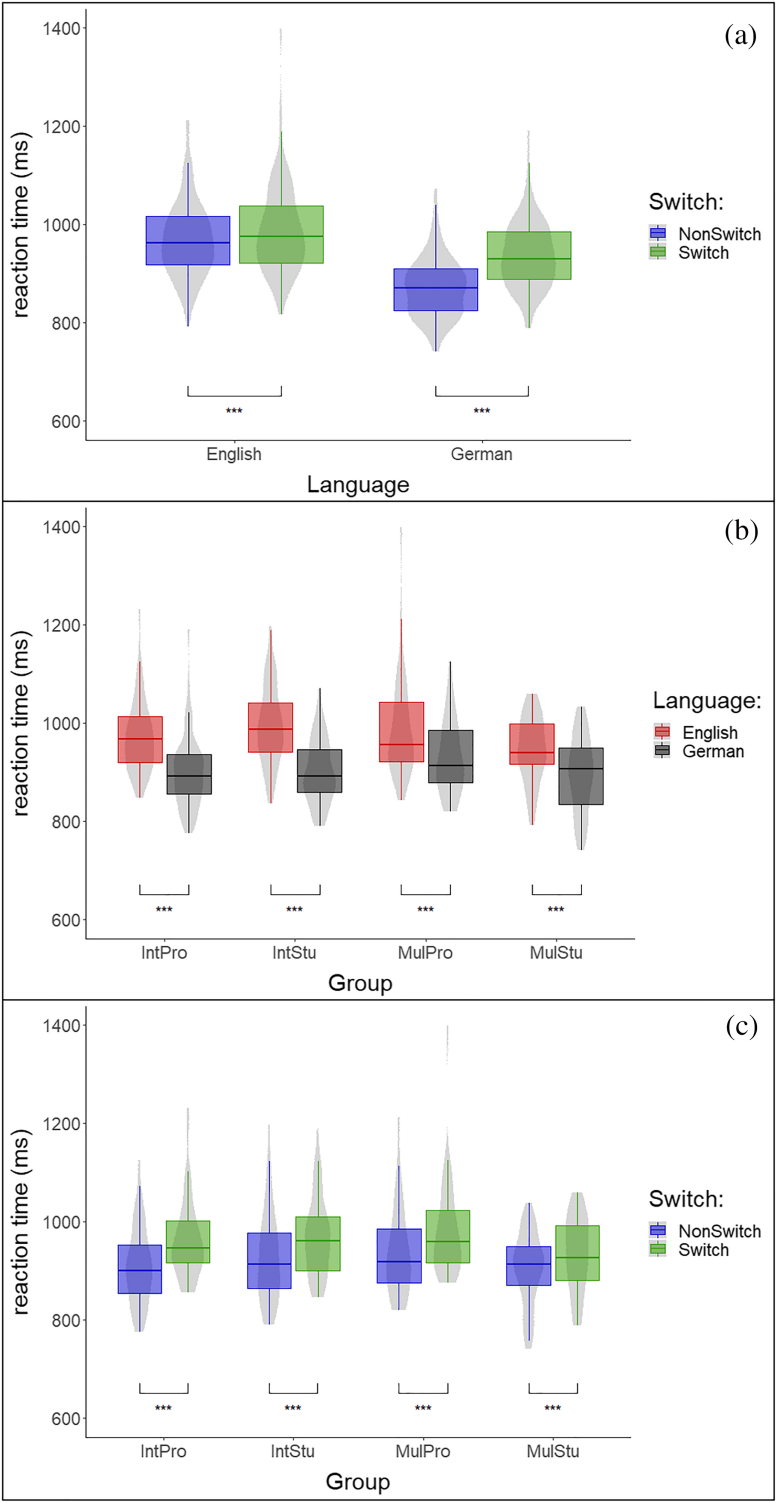
(a) Depiction of the ‘Language’ x ‘Switch’ interaction for the RT data. The difference between switch (green) and non‐switch trials (blue) was highly significant in both languages, whereas the condition difference was most pronounced in German trials. (b) Depiction of the ‘Group’ x ‘Language’ interaction. All four groups showed significantly longer RTs for English (red) compared to German (black) trials. Only the additional comparison of the difference in RTs between the group with the smallest (MulStu) and the highest (IntStu) values reached significance. (c) Depiction of the ‘Switch’ x ‘Group’ interaction effect. The comparison of switch (green) and non‐switch (blue) trials yielded a significant result in all groups. Furthermore, as depicted by the ‘Language’ x ‘Group’ interaction, only the post‐hoc comparison of IntPros showing the maximal difference between the conditions and MulStu with the minimal difference reached significance. Abbreviations: IntPro, professional SIs; IntStu, SI trainees; MulPro, foreign language teachers; MulStu, anglistics students, ***p < 0.001, **p < 0.01, *p < 0.05

The RT data was evaluated using a 2 × 2 × 4 ANOVA (2 ‘Language’ × 2 ‘Switch’ × 4 ‘Group’). This procedure yielded significant main effects of ‘Language’ (*F*_[1,85]_ = 229.404, *p* < .001, η^2^_G_ = 0.205) and ‘Switch’ (*F*_[1,85]_ = 272.387, *p* < .001, η^2^_G_ = 0.080) as well as significant ‘Language’ x ‘Switch’ (F_[1,85]_ = 85.728, *p* < .001, η^2^_G_ = 0.024), ‘Group’ x ‘Language’ (*F*_[3,85]_ = 3.925, *p* = .011, η^2^_G_ = 0.013) and ‘Group’ x ‘Switch’ (*F*_[3,85]_ = 2.837, *p* = .043, η^2^_G_ = 0.003) interaction effects. All significant effects are summarized in Figure 3.

For the main effect of ‘Language’, German words were recognized faster than English ones (G = 904 ms, SD = 73.5; E = 978 ms, SD = 84.7). Furthermore, the main effect of ‘Switch’ originated from faster RTs in response to non‐switch trials compared to switch trials (non‐switch = 920 ms, SD = 85.3; switch = 963 ms, SD = 84.5). Even though in both languages an effect of ‘Switch’ was found (English: *t*_(170)_ = 5.269, *p* < .001; German: *t*_(170)_ = 17.403, *p* < .001), the ‘Language’ x ‘Switch’ interaction originated from the fact that the RTs in switch and non‐switch trials were more similar in the English compared to German language (*t*_(85)_ = 8.804, *p* < .001, Figure 3). The significant ‘Group’ x ‘Language’ interaction (Figure 3) was related to a smaller RT difference between English and German trials in MulStu compared to IntStu (*t*_(85)_ = 3.202, *p* = .010). In fact, even though in all groups the comparison between the RTs to English versus German trials reached significance (IntPro: *t*_(85)_ = 7.928, *p* < .001; IntStu: *t*_(85)_ = 11.315, *p* < .001; MulPro: *t*_(85)_ = 5.474, *p* < .001; MulStu: *t*_(85)_ = 4.509, *p* < .001), only the contrast between the IntStu showing the largest language difference (95.7 ms) compared to the MulStu with the smallest difference (50.7 ms) reached significance. Finally, to disentangle the ‘Group’ x ‘Switch’ interaction shown in Figure 3, in a first step, we compared the condition differences within each group. This procedure testified that the ‘Switch’ effect was present in all groups (IntPro: *t*_(85)_ = 10.925, *p* < .001; IntStu: *t*_(85)_ = 9.011, *p* < .001; MulPro: *t*_(85)_ = 7.305, *p* < .001; MulStu: *t*_(85)_ = 5.195, *p* < .001). In a second step, we compared the estimates of the differences in RTs between the conditions of every group with each other. Only the contrast between IntPro (53 ms) and MulStu (31.2 ms) reached significance (*t*_(85)_ = 2.830, *p* = .029).

**FIGURE 4:**
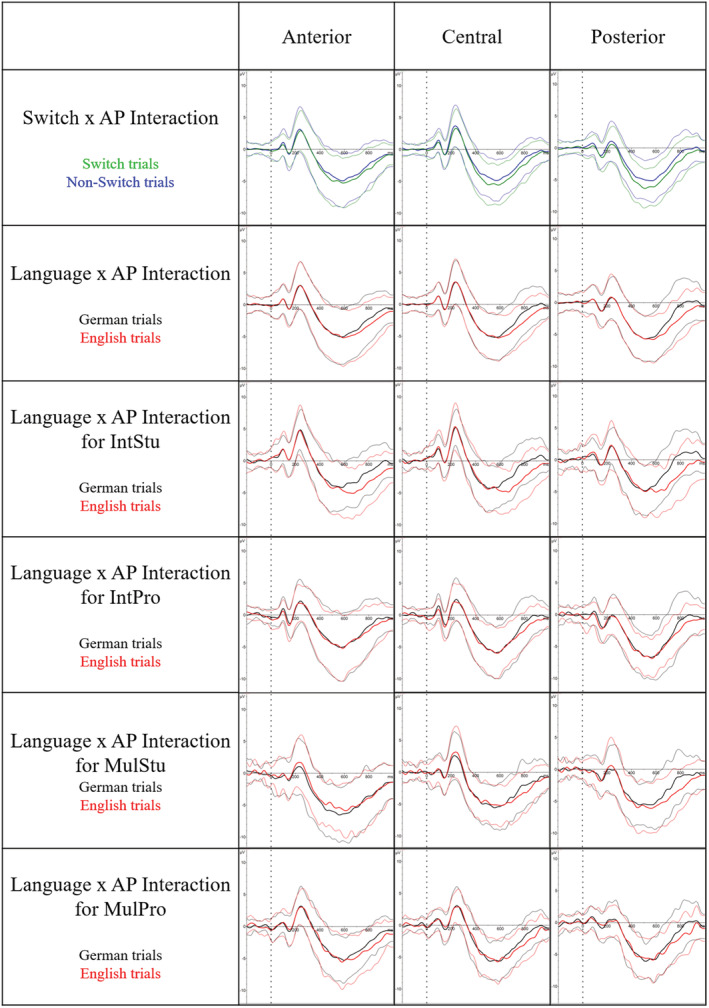
ERPs are shown for the anterior, central and posterior electrode clusters. On the top row, ERPs of switch trials (green) are shown together with ERPs of non‐switch trials (blue). As shown in the figure, the two conditions differed the most in the posterior cluster. The ERPs of the German (black) and English (red) trials depicted for the three clusters are in the second row. The difference between the two languages was largest at posterior sites. To disentangle the three‐way interaction of ‘Group’ x ‘Language’ x ‘AP’, the ERPs of the two languages, namely, German (black) and English (red), are depicted separately for each group. While for the three groups IntPro, IntStu and MulPro no evidence for mean N400 amplitude differences between both languages was found at the three electrode clusters, in MulStus the N400 differences between the two languages increased from anterior to central and posterior sites. All ERPs are visualized in bold, and the respective standard errors in thin lines. Abbreviations: IntPro, professional SIs; IntStu, SI trainees; MulPro, foreign language teachers; MulStu, anglistics students

The 2 × 2 × 4 × 3 ANOVA (2 ‘Language’ × 2 ‘Switch’ × 4 ‘Group’ × 3 ‘AP’) computed on mean N400 amplitudes yielded main effects of ‘Language’ (*F*_[1,85]_ = 6.484, *p* = .013, η^2^_G_ = 0.008) and ‘Switch’ (*F*_[1,85]_ = 13.723, *p* < .001, η^2^_G_ = 0.013) as well as significant ‘Language’ x ‘AP’ (*F*_[2,170]_ = 9.478, *p* = .001, η^2^_G_ = 0.001), ‘Switch’ x ‘AP’ (*F*_[2,170]_ = 12.076, *p* < .001, η^2^_G_ = 0.001) and ‘Group’ x ‘Language’ x ‘AP’ interaction effects (*F*_[6,170]_ = 3.718, *p* = .009, η^2^_G_ = 0.001). The main effect of ‘Language’ originated from the fact that English words elicited more negative N400 amplitudes than German ones (E = −3.58 μV, SD = 3.21; G = −3.04 μV, SD = 3.38). Otherwise, the main effect of ‘Switch’ was related to larger N400 amplitudes in response to switch compared to non‐switch trials (switch = −3.65 μV, SD = 3.22; non‐switch = −2.96 μV, SD = 3.36). All these significant effects are depicted in Figure 4.

As reported above, we also revealed three interaction effects, namely ‘Language’ x ‘AP’, ‘Switch’ x ‘AP’ and ‘Group’ x ‘Language’ x ‘AP’. The ‘Language’ x ‘AP’ interaction originated from the fact that English words elicited more negative N400 deflections than German words at central (*t*_[95]_ = 2.033, *p* = .045) and posterior (*t*_[95]_ = 3.092, *p* = .003) electrode sites. Furthermore, when comparing the language differences at the three electrode sites between each other, the contrast between the anterior and the central pool did not reach significance. Otherwise, the comparison between the central and posterior cluster (*t*_(170)_ = 2.577, *p* = .029) and between the anterior and posterior ones (*t*_(170)_ = 4.570, *p* < .001) yielded significant results, indicating that the difference between the two languages was most pronounced at posterior electrodes. The same procedure was also used to shed light on the ‘Switch’ x ‘AP’ interaction effect. Here, at all three electrode sites a difference between the two conditions was detected (anterior: *t*_(100)_ = 2.174, *p* = .032; central: *t*_(100)_ = 3.377, *p* = .001; posterior: *t*_(100)_ = 4.555, *p* < .001), with more negative N400 mean amplitudes in the ‘switch’ compared to the ‘non‐switch’ condition. To further inspect whether the condition differences were comparable in all clusters, we compared the estimates of the condition differences at the three electrode clusters. These comparisons revealed that the condition differences increased along an anterior–posterior gradient (anterior‐central: *t*_(170)_ = 2.431, *p* = .042; central‐posterior: *t*_(170)_ = 2.380, *p* = .048; anterior–posterior: *t*_(170)_ = 4.810, *p* < .001). Finally, the ‘Group’ x ‘Language’ x ‘AP’ interaction was further inspected by comparing the estimates of language differences with each other at the three electrode sites for every group. For the groups IntPro, IntStu and MulPro, the difference in mean N400 amplitudes between English and German words was not significantly different at the three electrode locations. On the other hand, in the MulStu the language differences in terms of N400 amplitudes differed between the electrode clusters (anterior‐central: *t*_(32)_ = 3.028, *p* = .013; anterior–posterior: *t*_(32)_ = 5.950, *p* < .001; central‐posterior: *t*_(32)_ = 2.933, *p* = .017).

We calculated an additional 2 × 2 × 4 × 3 ANOVA (2 ‘Language’ × 2 ‘Switch’ × 4 ‘Group’ × 3 ‘AP’) with N400 latency measures. This analysis yielded a main effect of ‘Language’ (*F*_[1,85]_ = 10.791, *p* = .001, η^2^_G_ = 0.016), a main effect of ‘AP’ (*F*_[2,270]_ = 69.713, *p* < .001, η^2^_G_ = 0.066), a ‘Switch’ x ‘Language’ interaction (*F*_[1,85]_ = 14.275, *p* < .001, η^2^_G_ = 0.016) and a ‘Switch’ x ‘Language’ x ‘AP’ interaction effect (*F*_[2,170]_ = 6.388, *p* = .005, η^2^_G_ = 0.004). As reflected in the main effect of ‘Language’, German words (M = 563 ms, SD = 110) elicited a faster N400 peak than English words (M = 587, SD = 115). In addition, the N400 peaked earlier at the posterior compared to central (*t*_(170)_ = 5.374, *p* < .001), and anterior locations (*t*_(170)_ = 11.113, *p* < .001). Furthermore, N400 latencies at central sites were smaller than at anterior sites (*t*_(170)_ = 5.740, *p* < .001). To disentangle the ‘Language’ x ‘Switch’ interaction effect, we compared the N400 latency switch effects separately for each language. This procedure resulted in a significant switch‐nonswitch difference in English (*t*_[165]_ = 3.332, *p* = .001) but not in German trials (*t*_[165]_ = 1.920, *p* = .057). Finally, the ‘Language’ x ‘Switch’ x ‘AP’ interaction showed that EE‐GG (central: *t*_(315)_ = 3.753, *p* = .001; posterior: *t*_(315)_ = 5.367, *p* < .001), EE‐GE (central: *t*_(367)_ = 3.080, *p* = .012; posterior: *t*_(367)_ = 4.735, *p* < .001), and EE‐EG trials (central: *t*_(310)_ = 3.017, *p* = .015; posterior: *t*_(310)_ = 3.185, *p* = 0.009) only significantly differed at central and posterior electrode sites.

## 4 DISCUSSION

### 4.1 General discussion

In the present study, we combined behavioural and EEG measurements to assess lexical access and code switching in multilinguals with different degrees of expertise. In line with previous studies, we were able to replicate the lexical status effect, which was reflected by more accurate and faster responses as well as smaller N400 amplitudes to words compared to pseudowords (Chwilla et al., 1995; Friedrich et al., 2006; Holcomb & Neville, 1990; Hut et al., 2017; López Zunini et al., 2020; Rodriguez‐Fornells et al., 2002). These results corroborate previous findings and support the notion that words are processed easier than pseudowords because they can be directly mapped onto the mental lexicon, whereas the processing of pseudowords requires a deeper lexical search and a greater computational capacity at the neural level (Friedrich et al., 2006; Specht et al., 2003).

The evaluation of lexical access in the two languages, namely, L1 (German) and L2 (English), revealed a lower accuracy along with longer RTs and larger N400 amplitudes while processing L2 compared to L1 trials. Otherwise, the comparison between switch and non‐switch trials showed that switch trials were generally associated with reduced accuracies, longer RTs, and increased N400 amplitudes compared to non‐switch trials. Furthermore, the difference between switch and non‐switch trials in terms of accuracy was larger in the English (L2) than the German (L1) language, whereas the discrepancy in RTs was more pronounced in the German language. Finally, the group comparisons also indicated a worse accuracy in the student groups, especially in response to English trials (‘Language’ x ‘Group’ interaction). However, this effect of accuracy was not reflected in RTs and N400 metrics. These results will be discussed in more detail in the following sections.

### 4.2 The influence of L1 and L2

The behavioural and EEG data of the bilingual auditory lexical decision task clearly replicated previous findings showing that the processing of L2 is more demanding than L1 (i.e., Hut et al., 2017; Mosca & De Bot, 2017). In our sample, these effects were mirrored by lower accuracy values, longer RTs as well as more negative N400 amplitudes and longer N400 peak latencies for L2 compared to L1 trials. Such a result was not unexpected since all participants were late multilinguals who acquired L2 at school and were characterized by lower L2 proficiency and exposure compared to L1. Furthermore, it is generally acknowledged that foreign languages rely on the same neural infrastructure underlying L1 but with important computational differences between native and non‐native languages (Hernandez & Meschyan, 2006; Hut et al., 2017; Perani & Abutalebi, 2005; Rodriguez‐Fornells et al., 2005, 2009). In particular, L2 and non‐native language processing in general need more cognitive resources that are necessary for accessing and controlling the weaker language and avoiding interferences from the more dominant L1 (Perani & Abutalebi, 2005; Rodriguez‐Fornells et al., 2005).

### 4.3 Language switch effect

In our study, switch trials were reflected by increased N400 amplitudes, longer RTs and lower accuracies than non‐switch trials, suggesting that code switching is generally associated with higher processing costs. In a previous EEG study, Hut et al. (2017) provided evidence showing that in balanced bilinguals with two L1s, switching between these two languages did not increase N400 amplitudes compared to non‐switch L1 trials. However, switching from a later learned L2 into both L1s, but not the change from L1 to L2, resulted in larger N400 amplitudes compared to non‐switch L1 trials in both languages. Our EEG results are somewhat in contrast with those of Hut and colleagues in that our data consistently revealed processing costs that were independent of code‐switching direction. Nevertheless, we also noticed a discrepancy in behavioural indices as a function of switching direction (i.e., ‘Switch’ x ‘Language’ interaction effects). In fact, the accuracy data showed that the difference between switch and non‐switch trials was more pronounced in the English compared to the German language, whereas the RT data pointed in the opposite direction. The notion that the N400 component was not sensitive to track the switching direction but we still revealed a dissociation of behavioural indices (i.e., accuracy and RTs) was somehow unexpected and is therefore not easy to explain. Based on the IC model (Green, 1986, 1998), the larger RT differences in the German (EG vs. GG) compared to the English switch trials (GE vs. EE) support the idea that backward switches from L2 to L1 were costlier because during the processing of the weaker L2, the more dominant L1 had to be inhibited, and such an inhibition has to be suppressed for enabling lexical access in L1. Otherwise, the accuracy data indicating stronger differences between switch and non‐switch trials in the English compared to the German language might be explained by the BIA model (Dijkstra & Van Heuven, 1998; Grainger & Dijkstra, 1992; van Heuven et al., 1998). In fact, in line with this theoretical framework, one may infer that the processing of the dominant L1 activates more phonological neighbours, leading to a stronger activation of the German language node, which in turn inhibits lexical access in L2. Hence, in order to switch from L1 to L2, L2 inhibition has to be overridden to enable lexical access. Furthermore, the fact that L2 was the weaker language might also explain the higher sensitivity of accuracy metrics to measure forward (i.e., from L1 to L2) switching costs. In particular, based on both EEG and behavioural data showing higher processing costs for L2, we might speculate that even in highly proficient L2 users, inhibition of L1 during a code‐switching task seems to be less efficient than the inhibition of L2. We conclude that a later age of acquisition in association with a lower proficiency and exposure is associated with switching costs that can reliably be captured by both behavioural and electrophysiological indices. This perspective is also in line with previous studies showing that age of acquisition and proficiency, but also the degree of exposure, have an influence on L2 processing (e.g., Consonni et al., 2013; Perani & Abutalebi, 2005; Perani et al., 2003; Wartenburger et al., 2003).

The somewhat divergent results of Hut et al. (2017) can also be explained by the different experimental designs used in our study. In fact, Hut et al. (2017) used a semantic categorization task, whereas our participants completed a lexical decision task. In this context, the main difference between the two procedures is that in our study, the participants did not have to access the meaning of the words but just to assess the word form and map it onto the mental lexicon. Hence, our results complemented the previous findings of Hut et al. (2017) in showing that switching costs are not only manifested at the semantic level of word processing but also at the lexical level. Another difference is that in the study of Hut et al. (2017), only 18% of trials per language were targets, resulting in less statistical power. It is also noteworthy to mention that it has been claimed that using a simple lexical decision task relying on the recognition of the lexical status of words would minimize code‐switching effects compared to more demanding tasks where the participants have to recognize the language corresponding to the words (Jackson et al., 2004). Furthermore, previous studies suggested that code‐switching effects depend upon the sensory modality involved, with larger costs in the visual than in the auditory modality (i.e., Declerck et al., 2015; López Zunini et al., 2020). In sum, even though most previous studies used visual stimuli to examine code switching during receptive tasks, our results corroborate that switching costs can also reliably be measured during auditory lexical decision tasks and captured by both behavioural and electrophysiological indices. Finally, our results did not indicate a typical speed‐accuracy trade‐off. In fact, German trials were answered with higher accuracy than English trials and were also reflected in faster response times. Furthermore, non‐switch trials were associated with higher accuracy as well as faster RTs compared to switch trials. This specific pattern of results highlights that non‐switch trials in L1 were tackled with high precision and high speed.

### 4.4 The effect of expertise in language switching

In the present work, we hypothesized that for individuals who are highly trained to switch between languages, namely, professional SIs, we would find evidence for reduced switching costs, at least in the trained language direction (i.e., from English to German). However, contrary to our expectation, the four groups did not differ in terms of N400 amplitudes. In contrast, the evaluation of RTs yielded a ‘Group’ x ‘Switch’ interaction effect, which was associated with larger RT differences between switch and non‐switch trials in professional SIs. Furthermore, the accuracy metrics revealed a main effect of ‘Group’, with more correct responses in professional SIs compared to the two groups of students. Even though these results were unexpected and somewhat surprising, the behavioural data demonstrated that professional SIs could reach high accuracies without a loss of speed. Interestingly, there is evidence indicating that older participants tend towards slower but more accurate responses in tasks relying on speed and accuracy (Salthouse, 1979). This finding was partly reflected in our data, with IntPros scoring more accurately and being older than both groups of students. On the other hand, RTs did not differ between these groups, and therefore, no evidence for a prioritization of accuracy over speed was present in IntPros. Such a perspective could be explained by the fact that in their daily work setting SIs have to provide accurate interpretations while maintaining a high processing speed.

A closer look at the groups' composition might explain the reason for not having detected between‐group differences in the EEG metrics. In fact, in this study, we deliberately recruited homogeneous groups of multilingual participants with a high L2 proficiency level. Furthermore, all groups used L2 in their everyday lives and learned English during childhood at the age of about 10 years. Interestingly, in a previous fMRI study, Consonni et al. (2013) measured two groups of bilinguals who were comparable in proficiency and language exposure but differed in age of acquisition. Notably, both groups of bilinguals demonstrated a complete neural overlap between L1 and L2 during sentence comprehension, suggesting the recruitment of the same neural network when proficiency and exposure are at a high level. The second line of interpretation is that SIs do not show an advantage in lexical processing but only at the semantic level. This is at least supported by a previous study by Elmer et al. (2010). The authors used a semantic decision task (i.e., congruent and incongruent word pairs) and uncovered larger N400 responses in SIs compared to control participants in response to incongruent word pairs. Furthermore, already in the 90s, Fabbro et al. (1991) reported an advantage of SIs in detecting semantic errors during a dichotic listening task. Previous studies have also repeatedly shown that the benefits of SIs seem to be very specific in nature and task‐dependent (i.e., Christoffels et al., 2006; Dottori et al., 2020; García, 2014; Hiltunen et al., 2016; Köpke & Signorelli, 2012; Morales et al., 2015; Stavrakaki et al., 2012). Hence, we might conclude that interpreting training does not benefit lexical processing and that expertise does not translate into word‐processing mechanisms other than semantics (Santilli et al., 2018). Otherwise, it is important to consider that in our study, we focused on language reception at the basic level of lexical processing, whereas simultaneous interpreting relies on both language reception and production, which are known to be related to distinct neural circuits (Hickok & Poeppel, 2007; Mosca & De Bot, 2017). In this regard, it is noteworthy to mention that the group of professional SIs we measured was principally trained in a mixed reception and production scenario but not in a solely receptive switching situation. This further implies that all groups were similarly experienced in hearing language switches, and this attitude was possibly also reflected in our results showing no group differences in terms of mean N400 amplitudes. Therefore, we did not find evidence for transfer effects in SI from interpreting training to switching mechanisms in a hearing‐only situation. This is also in line with previous findings indicating that advantages of interpreting training are closely tied to interpretation‐like scenarios (Köpke & Signorelli, 2012). With these considerations in mind, it is possible that larger between‐group differences would emerge when analysing code‐switching mechanisms during language production tasks or in experimental conditions requiring the interplay between language reception, language production and code switching. Finally, it is essential to mention that during simultaneous interpretation, code‐switching mechanisms are foreseeable and always in the same language direction, whereas in a lexical decision task, such changes are randomly distributed. Therefore, future studies should try to examine code‐switching mechanisms under more ecologically valid experimental conditions.

Taken together, our results have several implications. First, we provided evidence that the age of acquisition is of importance for describing switching costs between two languages. Despite demonstrating a high L2 proficiency as well as a high L2 exposure, our sample showed considerable switching costs, which accentuate the importance of the effect of age of acquisition. Second, with the implementation of an auditory lexical decision task, we were able to generalize the word‐pseudoword effect, the effect of language and the effect of language switching previously described in the context of visual tasks. Third, since we could not find an effect of interpreting training in a task relying on lexical processing, we provided evidence that advantages of simultaneous interpreting training seem to be task‐specific.

### 4.5 Limitations

The first limitation of this study is the small sample size of the groups we measured in association with the relatively noisy EEG data due to muscle artefacts that led to a relatively low statistical power. Furthermore, a modulation of the language exposure spectrum of the participants could provide additional information regarding the influence of this variable on lexical access and code switching. This could, for example, be done by including at least one group of bilinguals who do not use English (L2) in their daily lives but have a similar proficiency level. In our study, we manipulated both the languages and the lexical status but only used mid‐frequent words. A word frequency manipulation could shed more light on the effects of exposure on auditory language reception processes of highly proficient bilinguals. In addition, including different stimulus features such as cognates or phonetic and semantic neighbours might provide interesting additional insights. Future studies could also compare switching costs between foreseeable and unforeseeable language conditions, for example, using high and low constraint sentences.

## 5 CONCLUSIONS

In the present work, we examined lexical access and code‐switching mechanisms in different groups of highly proficient bilinguals, namely, professional SIs, trainee interpreters, foreign language teachers, and Anglistics students. Using an auditory lexical decision task, we replicated previous findings showing that lexical access in L2 is more demanding than in L1. Most notably, we also provided evidence that code‐switching in the auditory domain was generally associated with processing costs as reflected by lower accuracies, longer RTs and larger N400 amplitudes, irrespective of language direction. Finally, we also complemented previous findings in the domain of interpreting studies by showing that the expertise of SIs in code switching is not mandatorily manifested at the lexical but possibly only at the semantic level.

## CONFLICT OF INTEREST

The authors declare no potential sources of conflict of interest.

## AUTHOR CONTRIBUTIONS

MB, MK and SE designed the experiment. MB and MK collected the data. MB analysed the data and prepared the original draft. MB, MK, SE and LJ reviewed and edited the paper for submission. SE and LJ supervised the project.

### PEER REVIEW

The peer review history for this article is available at https://publons.com/publon/10.1111/ejn.15786.

## Supporting information


**Table S1:** List of stimuli used for the lexical decision task
**Table S2:** List of ANOVA outputs including residual sum of squares not reported in the main text for accuracy values.
**Table S3:** List of ANOVA outputs including residual sum of squares not reported in the main text for reaction time values.
**Table S4:** List of ANOVA outputs including residual sum of squares not reported in the main text for N400 mean amplitudes.

## Data Availability

Data are available upon request to the authors.

## Footnotes

^1^

https://ec.europa.eu/eurostat/statistics-explained/index.php?title=Foreign_language_skills_statistics (checked 12/2021; data from 2016).

^2^
E: https://corpora.uni-leipzig.de/en?corpusId=eng-uk_web_2012; G: https://corpora.uni-leipzig.de/en?corpusId=deu_newscrawl_2011
