# Supplementary material for: Tracking lexical access and code switching in multilingual participants with different degrees of simultaneous interpretation expertise
Source: Eur J Neurosci. 2022 Aug 9;56(6):4869–88. doi: 10.1111/ejn.15786 (PMC9544540; doi:10.1111/ejn.15786)
Supplement: Supplementary file 1 — Table S1: List of stimuli used for the lexical decision task Table S2: List of ANOVA outputs including residual sum of squares not reported in the main text for accuracy values. Table S3: List of ANOVA outputs including residual sum of squares not reported in the main text for reaction time values. Table S4: List of ANOVA outputs including residual sum of squares not reported in the main text for N400 mean amplitudes. [file EJN-56-4869-s001.pdf]

SUPPLEMENTARY MATERIAL

Table S1: List of stimuli used for the lexical decision task

Category	English Words	German Words	English Pseudowords	German Pseudowords
Vehicle	Trolley	Auto	Stockmock	Vieruck
Vehicle	Metro	Kanu	Coatmet	Taucke
Vehicle	Lorry	Fahrrad	Baglor	Zwiene
Vehicle	Aircraft	Flugzeug	Ningtrom	Fenman
Vehicle	Railcar	Bagger	Nefree	Spramus
Vehicle	Rickshaw	Roller	Plantmus	Zwiead
Vehicle	Trailor	Mofa	Primock	Zenschirr
Vehicle	Wagon	Kutsche	Rabmod	Kothrin
Vehicle	Subway	Traktor	Swalpen	Daucken
Vehicle	Rocket	Panzer	Sunpamp	Jogschu
Fruits and Nuts	Walnut	Apfel	Protsilp	Schrauschi
Fruits and Nuts	Lemon	Birne	Waistair	Zwieglet
Fruits and Nuts	Melon	Dattel	Soltrol	Schniewich
Fruits and Nuts	Chestnut	Feige	Pentwi	Rotkel
Fruits and Nuts	Almond	Kirsche	Shawmod	Scherich
Fruits and Nuts	Currant	Litschi	Rainshaw	Stiebrik
Fruits and Nuts	Raisin	Kaki	Mondon	Wilzlen
Fruits and Nuts	Rhubarb	Pfirsich	Peclean	Naleu
Fruits and Nuts	Berry	Pflaume	Pentrom	Fallze
Fruits and Nuts	Peanut	Traube	Anrobe	Slochen
Buildings	Prison	Schule	Bladrad	Bautin
Buildings	Resort	Kirche	Slitist	Bielip
Buildings	Warehouse	Fabrik	Winbut	Fenkohl
Buildings	Showroom	Werkstatt	Clorsot	Derfal
Buildings	Greenhouse	Rathaus	Wadbard	Tinseg
Buildings	Clinic	Labor	Oldress	Merseg
Buildings	Chapel	Palast	Trolfid	Bankja
Buildings	Cottage	Bunker	Garbad	Humwahl
Buildings	Airport	Scheune	Boncan	Pfante
Buildings	Castle	Bahnhof	Stirom	Rafus
Vegetables	Eggplant	Bohne	Soonber	Musner
Vegetables	Pumpkin	Gurke	Hedgesur	Melgen
Vegetables	Olive	Spargel	Cregar	Erlwob
Vegetables	Mushroom	Fenchel	Carcaft	Scheurol
Vegetables	Beetroot	Salat	Pawnsit	Reboh
Vegetables	Cabbage	Zwiebel	Prekey	Hudme
Vegetables	Carrot	Erbse	Swalroe	Pfanstik
Vegetables	Ginger	Rotkohl	Stocers	Truad
Vegetables	Radish	Spinat	Setpea	Tachret
Vegetables	Garlic	Rettich	Surfree	Scherfech
Clothings	Jumper	Stiefel	Monrot	Lerwan
Clothings	Stocking	Bluse	Mortist	Mannel
Clothings	Helmet	Hose	Beetbal	Schautich
Clothings	Slipper	Jacke	Dengren	Bluzeug
Clothings	Sweater	Mantel	Carftbeat	Weuka
Clothings	Wetsuit	Trikot	Piecot	Selwild
Clothings	Hoodie	Anzug	Sonchain	Senlau
Clothings	Apron	Korsett	Pigmal	Pfirber
Clothings	Waistcoat	Socke	Pilrag	Wolfser
Clothings	Gauntlet	Weste	Rhumon	Stattkla

Body parts	Elbow	Daumen	Trettle	Auber
Body parts	Ankle	Auge	Bobok	Nerla
Body parts	Forehead	Lippe	Leypan	Schrauseg
Body parts	Backbone	Gaumen	Floodbon	Settas
Body parts	Pupil	Lunge	Showgren	Wildhar
Body parts	Liver	Magen	Ladrich	Somga
Body parts	Muscle	Gehirn	Skatwrest	Seirot
Body parts	Bladder	Zunge	Mornal	Schalgenz
Body parts	Kidney	Nacken	Wearlpie	Tarmaul
Body parts	Shoulder	Nase	Wearloan	Maukasch
Kitchen utensils	Skillet	Gabel	Slipdol	Kuckdau
Kitchen utensils	Saucepan	Pfanne	Freabye	Waldat
Kitchen utensils	Teaspoon	Messer	Silearth	Walpfrei
Kitchen utensils	Mortar	Teller	Richset	Glafa
Kitchen utensils	Blender	Kessel	Twikey	Storschmitt
Kitchen utensils	Freezer	Ofen	Phincoat	Erkes
Kitchen utensils	Toaster	Waage	Shindrew	Traudat
Kitchen utensils	Cutter	Tasse	Caktard	Schlanaus
Kitchen utensils	Juicer	Schale	Bresteam	Nepa
Kitchen utensils	Steamer	Geschirr	Neyrel	Feitrom
Furniture	Table	Sessel	Railtrol	Lehmo
Furniture	Dresser	Schreibtisch	Ranthog	Sichtglib
Furniture	Candle	Kissen	Petpea	Daukan
Furniture	Hammock	Regal	Copair	Lamke
Furniture	Bookshelf	Hocker	Balcraft	Buntel
Furniture	Closet	Liege	Scrashle	Baune
Furniture	Cupboard	Spiegel	Lightclim	Kanbe
Furniture	Heater	Kerze	Albage	Tichsol
Furniture	Drawer	Schemel	Prirant	Scherad
Furniture	Wardrobe	Lampe	Denrobe	Scheuwe
Animals	Hedgehog	Katze		
Animals	Donkey	Hummel		
Animals	Lion	Bieber		
Animals	Reindeer	Kamel		
Animals	Rabbit	Widder		
Animals	Badger	Ziege		
Animals	Squirrel	Ratte		
Animals	Spider	Nashorn		
Animals	Monkey	Schlange		
Animals	Dolphin	Wildschwein		
Birds	Ostrich	Amsel		
Birds	Robin	Geier		
Birds	Peacock	Kuckuck		
Birds	Pigeon	Ente		
Birds	Penguin	Uhu		
Birds	Parrot	Meise		
Birds	Eagle	Elster		
Birds	Chicken	Eule		
Birds	Swallow	Rabe		
Birds	Turkey	Falke		
Tools	Chainsaw	Bohrer		
Tools	Ruler	Feile		
Tools	Workbench	Schraube		
Tools	Ladder	Nagel		
Tools	Jigsaw	Werkbank		
Tools	Mallet	Zirkel		
Tools	Pliers	Knarre		
Tools	Chisel	Hobel		

Tools	Scraper	Bolzen		
Tools	Gable	Schere		
Music	Guitar	Rhythmus		
Music	Trombone	Klavier		
Music	Trumpet	Geige		
Music	Ballad	Barock		
Music	Octave	Harfe		
Music	Cornet	Hymne		
Music	Bagpipe	Schlagzeug		
Music	Fiddle	Trommel		
Music	Quartet	Laute		
Music	Upbeat	Orgel		
Professions	Doctor	Tierarzt		
Professions	Dentist	Lehrer		
Professions	Surgeon	Bauer		
Professions	Artist	Soldat		
Professions	Actor	Kellner		
Professions	Soldier	Fischer		
Professions	Singer	Maler		
Professions	Driver	Maurer		
Professions	Tailor	Anwalt		
Professions	Cleaner	Richter		
Sports	Climbing	Tauchen		
Sports	Juggling	Fechten		
Sports	Hiking	Fussball		
Sports	Rowing	Reiten		
Sports	Bowling	Joggen		
Sports	Fishing	Turnen		
Sports	Hunting	Tanzen		
Sports	Skating	Segeln		
Sports	Skiing	Schwimmen		
Sports	Wrestling	Boxen		
Materials	Copper	Eisen		
Materials	Cotton	Wolfram		
Materials	Silver	Wolle		
Materials	Fibre	Platin		
Materials	Linen	Titan		
Materials	Timber	Seide		
Materials	Bamboo	Papier		
Materials	Rubber	Kaschmir		
Materials	Cardboard	Plastik		
Materials	Carbon	Leder		
Environment	Earthquake	Regen		
Environment	Eclipse	Wolke		
Environment	Lightning	Gletscher		
Environment	Thunder	Hagel		
Environment	Rainbow	Taifun		
Environment	Flooding	Nebel		
Environment	Autumn	Schauer		
Environment	Sunshine	Sommer		
Environment	Twilight	Schneesturm		
Environment	Sunrise	Orkan		

Table S2: List of ANOVA outputs including residual sum of squares not reported in the main text for accuracy values.

Code-switching accuracies:

	Effect	DFn	DFd	SSn	SSd	F	p	p<.05	ges
1	(Intercept)	1	85	3.326138e+02	0.11476368	2.463512e+05	6.150190e-149	*	0.999141292
2	Group	3	85	2.263305e-02	0.11476368	5.587740e+00	1.513379e-03	*	0.073365606
3	Switch	1	85	2.913891e-02	0.04891044	5.063964e+01	3.279115e-10	*	0.092503725
5	Language	1	85	1.542464e-01	0.07981154	1.642737e+02	1.463903e-21	*	0.350472284
4	Group:Switch	3	85	2.712482e-03	0.04891044	1.571314e+00	2.022930e-01		0.009399536
6	Group:Language	3	85	1.382006e-02	0.07981154	4.906164e+00	3.417806e-03	*	0.046115501
7	Switch:Language	1	85	9.618066e-03	0.04237798	1.929152e+01	3.216191e-05	*	0.032550464
8	Group:Switch:Language	3	85	6.717075e-04	0.04237798	4.490944e-01	7.185867e-01		0.002344240

Table S3: List of ANOVA outputs including residual sum of squares not reported in the main text for reaction time values.

Code-Switching reaction times:

	Effect	DFn	DFd	SSn	SSd	F	p	p<.05	ges
1	(Intercept)	1	85	3.153803e+08	1628530.12	1.646106e+04	4.393729e-99	*	0.9939797673
2	Group	3	85	7.035718e+04	1628530.12	1.224081e+00	3.060133e-01		0.0355246055
3	Switch	1	85	1.668289e+05	52059.89	2.723873e+02	3.056874e-28	*	0.0803223629
5	Language	1	85	4.925547e+05	182504.33	2.294036e+02	7.238544e-26	*	0.2049990292
4	Group:Switch	3	85	5.213598e+03	52059.89	2.837474e+00	4.282265e-02	*	0.0027219708
6	Group:Language	3	85	2.528185e+04	182504.33	3.924943e+00	1.124186e-02	*	0.0130625542
7	Switch:Language	1	85	4.747128e+04	47068.19	8.572793e+01	1.614675e-14	*	0.0242493154
8	Group:Switch:Language	3	85	1.151935e+03	47068.19	6.934228e-01	5.585714e-01		0.0006026926

Table S4: List of ANOVA outputs including residual sum of squares not reported in the main text for N400 mean amplitudes.

Code-switching N400s:

	Effect	DFn	DFd	SSn	SSd	F	p	p<.05	ges
1	(Intercept)	1	85	11684.306021	5907.00935	168.1334756	7.590304e-22	*	0.5380727934
2	Group	3	85	292.782581	5907.00935	1.4043496	2.471545e-01		0.0283605686
3	Switch	1	85	127.698796	790.97479	13.7228111	3.760022e-04	*	0.0125706405
5	Language	1	85	79.260361	1038.98542	6.4843361	1.268529e-02	*	0.0078397540
7	AP	2	170	22.605879	1243.03812	1.5458092	2.161200e-01		0.0022485799
4	Group:Switch	3	85	25.558638	790.97479	0.9155303	4.370198e-01		0.0025415408
6	Group:Language	3	85	32.267567	1038.98542	0.8799428	4.548931e-01		0.0032065349
8	Group:AP	6	170	65.765619	1243.03812	1.4990363	1.812189e-01		0.0065136644
9	Switch:Language	1	85	16.012175	823.86628	1.6520094	2.021767e-01		0.0015937572
11	Switch:AP	2	170	9.999526	70.38304	12.0762014	1.247449e-05	*	0.0009958898
13	Language:AP	2	170	6.910648	61.97304	9.4783958	1.251253e-04	*	0.0006884687
10	Group:Switch:Language	3	85	30.896831	823.86628	1.0625635	3.694184e-01		0.0030707386
12	Group:Switch:AP	6	170	1.921163	70.38304	0.7733815	5.918508e-01		0.0001914898
14	Group:Language:AP	6	170	8.131437	61.97304	3.7175955	1.687472e-03	*	0.0008099905
15	Switch:Language:AP	2	170	2.096655	94.56673	1.8845490	1.550572e-01		0.0002089781
16	Group:Switch:Language:AP	6	170	1.994882	94.56673	0.5976908	7.319114e-01		0.0001988362

$`Mauchly's Test for Sphericity`

	Effect	W	p	p<.05
7	AP	0.3487584	6.109923e-20	*
8	Group:AP	0.3487584	6.109923e-20	*
11	Switch:AP	0.3741337	1.167151e-18	*
12	Group:Switch:AP	0.3741337	1.167151e-18	*
13	Language:AP	0.3757150	1.393350e-18	*
14	Group:Language:AP	0.3757150	1.393350e-18	*
15	Switch:Language:AP	0.2675184	8.889053e-25	*
16	Group:Switch:Language:AP	0.2675184	8.889053e-25	*

$`Sphericity Corrections`

	Effect	GGe	p[GG]	p[GG]<.05	HFe	p[HF]	p[HF]<.05
7	AP	0.6056049	0.2194801690		0.6096522	0.2195258477	
8	Group:AP	0.6056049	0.2121830639		0.6096522	0.2118532130	
11	Switch:AP	0.6150567	0.0003071221	*	0.6194932	0.0002959212	*
12	Group:Switch:AP	0.6150567	0.5355335421		0.6194932	0.5363517194	
13	Language:AP	0.6156555	0.0013580250	*	0.6201168	0.0013207179	*
14	Group:Language:AP	0.6156555	0.0087351301	*	0.6201168	0.0085672969	*
15	Switch:Language:AP	0.5772067	0.1718354403		0.5801114	0.1717588135	
16	Group:Switch:Language:AP	0.5772067	0.6415190944		0.5801114	0.6423357227	
